# Grandmothers’ smoking in pregnancy is associated with a reduced prevalence of early-onset myopia

**DOI:** 10.1038/s41598-019-51678-9

**Published:** 2019-10-28

**Authors:** Cathy Williams, Matthew Suderman, Jeremy A. Guggenheim, Genette Ellis, Steve Gregory, Yasmin Iles-Caven, Kate Northstone, Jean Golding, Marcus Pembrey

**Affiliations:** 1 Centre for Academic Child Health, Population Health Sciences, Bristol Medical School, Oakfield House, Oakfield Grove, University of Bristol, Bristol, BS8 2BN UK; 2 MRC Integrative Epidemiology Unit, Bristol Medical School, Oakfield House, Oakfield Grove, University of Bristol, Bristol, BS8 2BN UK; 3 School of Optometry & Vision Sciences, Cardiff University, Maindy Road, Cardiff, CF24 4HQ UK; 4 ALSPAC, Oakfield House, Oakfield Grove, University of Bristol, Bristol, BS8 2BN UK

**Keywords:** Epigenetics, Epidemiology

## Abstract

Myopia (near sightedness) is the most common vision disorder resulting in visual impairment worldwide. We tested the hypothesis that intergenerational, non-genetic heritable effects influence refractive development, using grandparental prenatal smoking as a candidate exposure. Using data from the Avon Longitudinal Study of Parents and Children (ALSPAC), we found that the prevalence of myopia at age 7 was lower if the paternal grandmother had smoked in pregnancy, an association primarily found among grandsons compared to granddaughters. There was a weaker, non-sex-specific, reduction in the prevalence of myopia at age 7 if the maternal grandmother had smoked in pregnancy. For children who became myopic later (between 7 and 15 years of age) there were no associations with either grandmother smoking. Differences between early and late-onset myopia were confirmed with DNA methylation patterns: there were very distinct and strong associations with methylation for early-onset but not later-onset myopia.

## Introduction

Refractive eye development post-delivery is a strictly coordinated process: visual experience fine-tunes a developmental program of ocular growth towards an optimal match between the optical power of the eye and its axial length, normally resulting in sharp vision^1^. Failure to achieve or maintain this optimum leads to refractive errors including myopia (near sightedness). Myopia is the most common vision disorder worldwide and its prevalence is increasing^2^. For instance, in the United States the prevalence of myopia has increased from 25% to 44% of the adult (over 30) population^3^, and has reached more than 80% in young adults in some areas of South and Southeast Asia. Myopia characteristically emerges during school age (5–16 years of age). It is a complex ocular trait that results from an interplay of genes and environmental factors, many of which are poorly understood or currently unknown^4^.

Epidemiological and genetic studies have established that whilst myopia is heritable and known genetic variants increase risk, there are several well established and some less well established environmental exposures that are associated with myopia prevalence in a variety of settings and subjects^2,5^. In statistical models, myopia shows a complex Gene × Education interaction whereby the risk is multiplicative rather than additive in individuals with both a high genetic predisposition (i.e. those with a high genetic risk score) and a higher-than-average number of years in education, but the causal pathways are unclear^6^. Excessive close work, such as reading in childhood is a risk factor for myopia^7^, as is insufficient time outdoors^8–10^, although it should be noted that these associations have not been found consistently.

The risk (odds ratio) of developing myopia is increased approximately 2–5 fold among children with two myopic parents compared to children with no myopic parents^11–13^. Twin estimates of heritability are consistently high and suggest that genetic and shared environmental factors contribute as much as 70–90% to the inter-individual variation in refractive error^14,15^. Yet to date (in line with many complex traits), genome wide association studies show that common DNA variants account for less than 10% of the variance in refractive error^16^. The search is on for associated rare DNA variants using larger samples^17^ and copy number variants (CNVs) are also being assessed^18^. However, no matter how the contribution of DNA variants is estimated, only about 30% of the 70–90% heritability is currently accounted for^19^; thus other forms of gametic inheritance may play a role.

Increasing evidence from mammalian experiments suggests that there are exposures in early life, for example: environmental toxins, dietary challenges or psycho-social stress that can be transmitted biologically in some way to the next or subsequent generations. If the germ cells of the next generation(s) are exposed directly this is known as intergenerational, or transgenerational if not. A prime mediating candidate is inter/trans-generational epigenetic inheritance^20^. As well as environmentally-induced changes to the egg or sperm epigenome, sperm can also carry induced non-coding RNAs (ncRNAs). In mice, it has been demonstrated that these ncRNAs can be transferred to maturing post-testis sperm from the somatic epididymal epithelial cells^21^, in breach of August Weismann’s historic ‘barrier’. Human cross-generational observations are in line with these animal studies. Although the mechanisms involved remain unclear^22^, the associations are increasingly difficult to explain in terms of classical genetic inheritance.

Building on the original Őverkalix study of the association of food supply of paternal grandparents during their mid-childhood on their grandchildren’s longevity by Bygren and colleagues^23^, we collaborated in showing that such associations were sex-specific (but not sex-limited)^24^. It should also be noted that inter/trans-generational associations need not result in ‘inheritance of acquired characteristics’ but they often do so. Developmental outcomes in the next generation can be in the opposite direction, and appear as an adaptation. We found this in our previous intergenerational studies of maternal smoking in pregnancy using the ALSPAC cohort: if the study mother did not smoke in pregnancy but the maternal grandmother did, against expectation (direct prenatal tobacco exposure leads to a smaller birth weight) the grandsons were larger at birth than if neither grandmother nor mother had smoked^25^. These grandsons had greater lean mass and cardiovascular fitness when followed into adolescence^26^, but there was no such outcome in granddaughters, illustrating that sex-specific intergenerational associations can also occur when the initial exposure is tobacco during fetal life. We subsequently showed that the maternal grandmother smoking in pregnancy (but not the mother) was associated with an increase in autistic traits in the granddaughters rather than the grandsons^27^.

Thus, as a result of our previous studies, for the present investigation we hypothesized that any association of myopia with either grandmother smoking in pregnancy would be sex-specific and more obvious if the mother herself did not smoke.

## Results

### Bias in attendance

Table 1 demonstrates the proportions of children attending the ALSPAC research clinic at which refractive error was assessed at age 7 years, according to the smoking history of their parents and grandparents. In all instances, the chance of the child/grandchild attending the clinic was reduced in children whose parents or grandparents had smoked. The differences were less for the fathers and grandfathers who smoked compared with the mothers and grandmothers, especially if the mothers and grandmothers had smoked in pregnancy. All these differences were highly significant (P < 0.001). In contrast there were only small differences between the likelihood of attendance between the boys and girls (54.0% and 56.1% respectively).

**Table 1:** The proportion of children attending for myopia testing at 7 years of age, according to the smoking history of their parents and grandparents.

Smoking history		Proportion (%) examined at 7
MGM	Ever	3785/7129 (53.1)
	Never	3246/5451 (59.5)
MGM	Prenatally	2372/4733 (50.1)
	Not prenatally	4627/7790 (59.4)
MGF	Ever	5188/9422 (55.1)
	Never	1758/1179 (59.9)
PGM	Ever	3153/5462 (57.7)
	Never	2589/4119 (62.9)
PGM	Prenatally	2281/4028 (56.6)
	Not prenatally	3439/5516 (62.3)
PGF	Ever	4188/6972 (60.1)
	Never	1146/1898 (60.4)
Mother	Ever	3309/6629 (49.9)
	Never	3990/6431 (62.0)
Mother	Prenatally	1085/2615 (41.5)
	Not prenatally	6252/10548 (59.3)
Father	Ever	3000/5412 (55.4)
	Never	2863/4421 (64.8)
Father	Prenatally	1482/2959 (50.1)
	Not prenatally	4291/6709 (64.0)

MGM = maternal grandmother; MGF = maternal grandfather; PGM = paternal grandmother; PGF = paternal grandfather.

### Myopia at age 7

Of the 7985 children examined at 7 years of age, 205 were categorized as myopic; however, 49 of these were not classified as being myopic at age 10 and were therefore omitted from the analysis (on the assumption that they had been exhibiting pseudomyopia at age 7 and were therefore ‘false positives’ at that age - see Methods section below). Thus, the estimated prevalence of myopia at age 7 was 1.97% [156/7936]. There was no difference in the prevalence of myopia between boys and girls [1.94% v 1.99%; P = 0.876].

### Myopia at age 15

After subtracting those who had already been identified as myopic at age 7, the number of children assessed as myopic at age 15 identified 806 children who developed myopia between ages 7 and 15 years (a prevalence of 16.5%). Of these 350 were boys and 456 girls.

### Relationships between prevalence of myopia by age 7, or developing between 7–15 years, and features of the study child’s grandparents

As shown in Table 2, the proportion of children with myopia identified in the research clinic at age 7 varied with the demographic characteristics and smoking history of the grandparents. For the maternal grandparents, there were associations with ethnicity, whether the maternal grandmother had ever smoked, especially if they had smoked in pregnancy, and whether or not the mother was the firstborn child of the maternal grandmother. For the paternal grandparents, apart from associations with the paternal grandmother not smoking, especially not smoking in pregnancy, there were associations with the paternal grandfather having been a smoker and being older at the time of the birth of the study father. These factors were all considered in a backward stepwise logistic regression analysis (see below). It was notable that there was no evidence for associations with either the educational achievements or the social backgrounds of the grandparents.

**Table 2:** Proportion (n) of children assessed as myopic at 7 according to features of their grandparents.

Variable	MGM	MGF	PGM	PGF
**Year of birth**				
Pre 1925	2.5% (17)	2.6% (30)	2.3% (16)	2.0% (21)
1925–1929	2.4% (23)	2.4% (26)	1.4% (10)	2.3% (17)
1930–1934	1.9% (27)	1.9% (26)	3.0% (27)	2.5% (20)
1935–1939	2.0% (30)	1.8% (23)	1.8% (14)	1.5% (10)
1940–1944	1.3% (16)	1.7% (14)	1.2% (7)	1.2% (4)
1945+	1.6% (14)	1.4% (6)	1.2% (3)	2.4% (3)
P	0.341	0.626	0.127	0.568
N	6636	6229	3905	3763
**Ethnic background**				
White	1.9% (133)	1.9% (130)	1.9% (109)	1.9% (108)
Non-white	4.2% (5)	5.2% (7)	2.5% (3)	2.2% (3)
P	**0.082**	**0.008**	0.635	0.843
N	7129	7101	5764	5752
**Education level**				
Lower	1.8% (62)	2.2% (66)	2.1% (61)	1.9% (50)
Higher	2.4% (49)	2.1% (44)	2.1% (33)	2.4% (42)
P	0.170	0.861	0.913	0.229
N	5440	5116	4454	4468
**Ever smoked**				
Yes	1.7% (63)	2.0% (102)	1.4% (44)	1.8% (74)
No	2.3% (75)	1.9% (34)	2.7% (69)	2.6% (30)
P	**0.053**	0.933	**<0.001**	**0.067**
N	7031	6946	5742	5334
**Age at birth of parent**				
<25 years	1.8% (44)	1.4% (16)	1.6% (25)	1.0% (8)
25–34	1.9% (64)	2.0% (74)	2.1% (57)	2.0% (55)
35+	2.3% (19)	2.5% (35)	2.2% (15)	2.5% (31)
P	0.475	0.107	0.201	**0.015**
N	6636	6229	4947	4769
**Parity**				
0	1.5% (36)	NA	1.8% (15)	NA
1+	2.2% (105)		2.4% (33)	
P	**0.080**		0.361	
	6999		2163	
**Smoked prenatally**				
Yes	1.4% (33)	NA	1.2% (28)	NA
No	2.2% (103)		2.5% (85)	
P	**0.018**		**0.001**	
N	6999		5720	
**Social group**				
P	0.551	0.140	0.799	0.542
N	4092	6078	3055	5347

[P values < 0.10 are in bold].

MGM = maternal grandmother; MGF = maternal grandfather; PGM = paternal grandmother; PGF = paternal grandfather.

The relationships observed for myopia at age 7 (Table 2) were not found for myopia at age 15 (after excluding the children who had myopia at 7). Indeed, at age 15 there were no associations with smoking in any grandparent, but there were differences associated with the ethnic background of both maternal and paternal grandparents, with children of non-white parents being at increased risk (Supplementary Table 1).

### Unadjusted and adjusted analyses related to maternal grandmother smoking in pregnancy

The unadjusted relationships between maternal grandmother smoking in pregnancy and myopia in the 7-year-old grandchild (Table 3) demonstrated an apparently protective association: OR = 0.62 [95% CI 0.41, 0.91]. The degree of association was similar for boys and girls (OR = 0.61 vs. OR = 0.62, respectively).

**Table 3:** The odds [95%CI] of the grandchild having myopia at age 7 if the maternal grandmother smoked.

Population	Unadjusted OR [95%CI]	Adjusted (A) OR [95%CI]	Adjusted (B) OR [95%CI]
*All children*	N = 6995	N = 6565	N = 6995
Boys + Girls	0.62 [0.41, 0.91]*	0.63 [0.41, 0.95]*	0.60 [0.40, 0.91]*
Boys only	N = 3557		
	0.61 [0.35, 1.08](*)	DNE	DNE
Girls only	N = 3438		N = 3261
	0.62 [0.36, 1.07](*)		0.60 [0.33, 1.07](*)
*Mother non-smoker*	N = 5953	N = 5609	N = 5953
Boys + Girls	0.57 [0.37, 0.89]*	0.59 [0.37, 0.94]*	0.57 [0.36, 0.90]*
Boys only	N = 3017		
	0.52 [0.27, 0.99]*	DNE	DNE
Girls only	N = 2936		
	0.62 [0.34, 1.14]	DNE	DNE
*Mother smoked*			
Boys + Girls	N = 1020		
	0.95 [0.36, 2.47]	DNE	DNE
Boys only	N = 531		
	1.29 [0.34, 4.85]	DNE	DNE
Girls only	N = 489		
	0.66 [0.16, 2.79]	DNE	DNE

The table shows the unadjusted and adjusted odds, for (a) all children; (b) for children whose mothers had not smoked in pregnancy, and (c) for children whose mothers had also smoked in pregnancy. Adjustments are: (A) for features of the grandparents’ background; (B) also including maternal vision impairments. (In bold are the adjusted results significant at P < 0.01).

DNE = did not enter; OR = odds ratio; AOR = adjusted odds ratio;

NS = P > 0.10; (*)P < 0.10; *P < 0.05; **P < 0.01; ***P < 0.001.

The association was no different when the analysis was restricted to children whose mothers *did not* smoke prenatally: OR = 0.57 [95% CI 0.37, 0.89] and was again similar in boys and girls (OR = 0.52 vs. OR = 0.62, respectively, P = 0.671).When the other features of maternal grandmother’s background were included in the analysis model, similar ORs were identified: for all children, AOR = 0.63 [95% CI 0.41, 0.95]; for children whose mothers did not smoke in pregnancy, AOR = 0.59 [95% CI 0.37, 0.94]. Further adjustment for maternal self-reported impaired visual acuity made little difference to these results (Table 3).

For children whose mothers *did* smoke in pregnancy, the association between myopia at age 7 and smoking in pregnancy by the maternal grandmother was close to that expected under the null hypothesis, OR = 0.95 [95% CI 0.36, 2.47]. However, the sample size for this analysis was approximately one third the size of that for the analysis of children whose mothers had not smoked in pregnancy, and the confidence interval was wide.

As with the demographic characteristics, the results were different when using myopia developing between 7 and 15 years as the outcome. There were no associations between the smoking history of the maternal grandmother and myopia developing between 7 and 15 years in her grandchildren (Supplementary Table 3)

### Unadjusted and adjusted analyses related to paternal grandmother smoking in pregnancy

For associations between the paternal smoking and her grandchild’s myopia (Table 4), the unadjusted ORs indicated strong ‘protective’ associations between paternal grandmother’s prenatal smoking and myopia at age 7 years for all children (OR = 0.49 [95% CI 0.32, 0.75]), and in boys (OR = 0.30 [95% CI 0.15, 0.62] but less so for girls (OR = 0.68 [95% CI 0.39, 1.18]). There was some evidence to suggest that the association with boys was significantly different from that found with girls (P = 0.080). The associations among the children whose mothers *did not* themselves smoke in pregnancy were even more marked: for all children, OR = 0.41 [95% CI 0.25, 0.67]; for boys, OR = 0.27 [95% CI 0.12, 0.60] (both results P < 0.001) and again less so for girls OR = 0.57 [95% CI 0.31, 1.05]. Adjustment for potential confounders only minimally changed the sizes of the associations (Table 4). However, no such associations were found for myopia developing between 7 and 15 years as the outcome (data not shown).

**Table 4:** The odds [95%CI] of the grandchild having myopia at age 7 if the paternal grandmother smoked.

Population	Unadjusted OR [95%CI]	Adjusted (A) OR [95%CI]	Adjusted (B) OR [95%CI]
*All children*	N = 5717	N = 4396	N = 4099
Boys + Girls	0.49 [0.32, 0.75]***	0.42 [0.25, 0.69]***	0.47 [0.28, 0.79]**
Boys only	N = 2886	N = 2424	N = 2478
	0.30 [0.15, 0.62]***	0.27 [0.12, 0.60]***	0.31 [0.14, 0.65]**
Girls only	N = 2831		N = 3261
	0.68 [0.39, 1.18]	DNE	0.60 [0.33, 1.07](*)
*Mother non-smoker*	N = 4952	N = 4200	N = 3627
Boys + Girls	0.41 [0.25, 0.67]***	0.43 [0.26, 0.71]***	0.45 [0.27, 0.78]**
Boys only	N = 2498	N = 2124	N = 2498
	0.27 [0.12, 0.60]***	0.26 [0.11, 0.61]**	0.30 [0.13, 0.68]**
Girls only	N = 2454		
	0.57 [0.31, 1.05](*)	DNE	DNE
*Mother smoked*			
Boys + Girls	N = 739		
	1.39 [0.44, 4.42]	DNE	DNE
Boys only	N = 377		
	0.71 [0.12, 4.31]	DNE	DNE
Girls only	N = 362		
	2.30 [0.44, 12.00]	DNE	DNE

The table shows the unadjusted and adjusted odds, for (a) all children; (b) for children whose mothers had not smoked in pregnancy, and (c) for children whose mothers had also smoked in pregnancy. Adjustments are: (A) for features of the grandparents’ background; (B) also including maternal vision impairments.

(In bold are the adjusted results significant at P < 0.01).

DNE = did not enter; OR = odds ratio; AOR = adjusted odds ratio; NS = P > 0.10;

*P < 0.10; *P < 0.05; **P < 0.01; ***P < 0.001.

### Associations between maternal smoking and myopia developing by age 7, or between 7–15 years

We investigated whether similar associations existed if the mother herself smoked in pregnancy. The mothers of the ALSPAC study children were asked about their history of smoking at five time points – ever, immediately prior to pregnancy, and in the first, second and third trimesters (Supplementary Table 3). Maternal smoking was associated with a marginal reduction in the risk of myopia at the P = 0.10 level, which was equally true for myopia at 7 and myopia at 15. There were no associations with partner smoking in pregnancy, or of the mother being exposed to environmental tobacco smoke.

### Associations between DNA methylation and myopia

**Figure 1:**
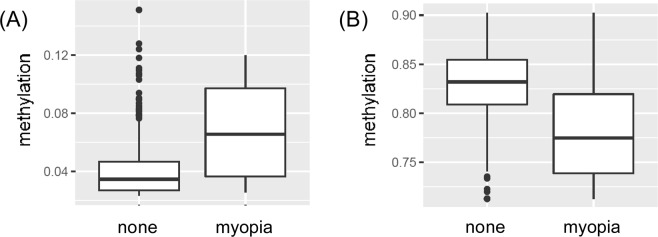
Myopia and DNA methylation. Cord blood DNA methylation of males with and without myopia at 7(p = 4.1 × 10^−8^) at CpG site cg03905867 in the *PAX6* gene (**A**). Peripheral blood DNA methylation level of 15–17 year-old males with and without myopia at 7 (p = 6.4 × 10^−5^) at CpG site cg13403566 (**B**), which is located less than 100 bp upstream of candidate gene *RASGRF1*.

Having observed differences in the relationships of myopia at 7 and myopia developing between 7–15, with grandparent demographics and smoking histories, we asked whether their molecular associations might also differ. To this end, we tested for associations between DNA methylation at approximately 450,000 CpG (cytosine and guanine nucleotides separated by a phosphate group) sites and myopia. Methylation levels were assessed in samples obtained at 3 age points: in cord blood collected at birth and in peripheral blood collected at ages 7 years and 15–17 years. Samples were available for only a subset of the study children (Supplementary Table 4); however, nearly all of the children in this subset had DNA methylation profiles at each of the three blood collection age-points (Supplementary Table 5). Several associations with myopia at 7 survived adjustment for multiple tests but none survived for myopia at 15 (Bonferroni adjusted p < 0.05; Table 5; Fig. 1a).

**Table 5:** Associations between myopia at age 7 and DNA methylation levels at CpG sites.

CpG site	Chr	Position	Gene	Age sample taken	Sex	Estimate	SE	P-value	n	Adjusted p-value	Potential genetic artefact*
cg26547698	16	89489058	ANKRD11	birth		−0.043	0.007	5.43E-09	862	0.003	yes
cg03905867	11	31821064	PAX6	birth	males	0.028	0.005	4.05E-08	421	0.019	no
cg06242000	3	183966155	ALG3	7	females	0.025	0.004	5.44E-08	465	0.026	yes
cg23680026	4	119200183	SNHG8	7	females	0.019	0.003	5.50E-09	465	0.003	no
cg12227341	2	99797610	MRPL30	15–17		0.007	0.001	5.47E-08	921	0.026	no
cg08656333	3	194218174	AX746839	15–17		−0.020	0.004	9.38E-08	921	0.044	no
cg12492276	12	109204605	SSH1	15–17		−0.056	0.010	3.14E-08	921	0.015	yes
cg02291011	1	111510137	LR1F1	15–17	males	−0.077	0.014	8.43E-08	445	0.040	yes
cg17241657	4	113152573	AP1AR	15–17	males	0.009	0.002	5.86E-08	445	0.028	no
cg23840823	4	1950185	WHSC1	15–17	males	0.099	0.018	1.04E-07	445	0.049	no
cg23249667	6	31515309	NFKBIL1	15–17	males	−0.003	0.000	1.90E-08	445	0.009	yes
cg05115182	10	99496889	ZFYVE27	15–17	males	0.020	0.004	6.95E-08	445	0.033	yes
cg19903001	10	70939225	SUPV3L1	15–17	males	0.059	0.011	1.05E-07	445	0.050	yes
cg06635871	12	7863853	DPPA3	15–17	males	0.080	0.014	3.79E-08	445	0.018	yes
cg00928894	16	188269	NPRL3	15–17	males	0.029	0.005	1.63E-08	445	0.008	no
cg24350392	17	37026147	LASP1	15–17	males	0.005	0.001	1.88E-08	445	0.009	no
cg16887862	1	1243669	ACAP3	15–17	females	0.014	0.003	9.19E-08	476	0.043	no
cg14673932	3	183872926	DVL3	15–17	females	0.009	0.002	2.14E-08	476	0.010	no
cg14119616	11	17099403	RPS13	15–17	females	0.008	0.001	9.57E-09	476	0.005	no
cg11451801	16	67970768	PSMB10	15–17	females	0.011	0.002	7.46E-09	476	0.004	no

Previous work has identified more than 150 genes and loci associated with myopia^16,28^. Before any analysis we selected nine of these known genes (Supplementary Table 6) – giving preference to those implicated as being imprinted or involved in gene-environment interactions – and tested whether they were enriched for CpG sites whose methylation level was associated with myopia at 7 or 15. We performed a look-up of the myopia summary statistics for the CpG sites near these genes among the 450 K CpG sites. Three associations survived adjustment for multiple tests, two with myopia at 7 and one with myopia at 15 (Bonferroni-adjusted p < 0.05 for 392 tests; Table 6). This included an association of myopia at 7 with DNA methylation at CpG site cg13403566 near *RASGRF1* (uncorrected p = 6.4 × 10^−5^; Fig. 1b).

**Table 6:** CpG sites associated with myopia near myopia gene candidates.

	Age samples taken	Sex	CpG sites	Chr	Position	Gene	P-value	Potential genetic artefact*
Myopia at 7	15–17	males	cg13403566	15	79357037	RASGRF1	6.36e-05	no
Myopia at 7	birth	males	cg02217063	16	6348709	RBFOX1	1.20e-05	no
Myopia at15	7		cg20414618	6	22136363	CASC15	9.57e-05	yes

P-values for these associations survived adjustment for multiple tests (Bonferroni-adjusted P < 0.05 for 392 tests).

We then compared the capacity of DNA methylation to predict myopia by constructing models of myopia in DNA methylation and evaluating their performance under 10-fold cross validation. Capacity was measured as the area under the receiver operating characteristic curve (AUC) of the model within each testing partition. Overall capacity was measured as the average AUC across all testing partitions. For myopia at age 7, average AUC was only slightly higher than for myopia at 15, but quite a bit higher (on average more than 0.1) when restricted to individual sexes (Table 7).

**Table 7:** Capacity of DNA methylation to predict myopia assessed under 10-fold cross validation and measured as mean and standard deviation of area under the receiver operating characteristic curve (AUC) across the 10 folds.

Age samples taken	Sex	Myopia at 7		Myopia at 15		Difference between 7 and 15
		Mean AUC	SD AUC	Mean AUC	SD AUC	p-value
Birth		0.64	0.11	0.57	0.04	0.17
	males	0.75	0.14z	0.60	0.06	0.019
	females	0.71	0.14	0.66	0.09	0.55
7y		0.62	0.11	0.57	0.09	0.20
	males	0.72	0.12	0.58	0.09	0.019
	females	0.85	0.14	0.56	0.05	0.0011
15–17y		0.60	0.12	0.58	0.06	0.71
	males	0.80	0.11	0.62	0.09	0.0041
	females	0.80	0.17	0.51	0.06	0.00088

P-values are calculated using the Wilcoxon rank-sum sum test comparing the AUC values across the 10 folds.

Finally, we asked how well DNA methylation associated with myopia at age 7 predicted myopia at 15, and vice versa. We evaluated this by constructing predictors as above for myopia at 7 but using the 10 CpG sites most strongly associated with myopia at 15 without cross-validation. We did the same for myopia at 15 using CpG sites associated with myopia at 7. In spite of omitting cross-validation, we generally observed AUCs near 0.5 indicating little or no predictive capacity (Table 8).

**Table 8:** Capacity of the top 9 CpG sites associated with myopia at 7y to predict myopia at 15–17y, and vice versa.

Age samples taken	Sex	Myopia at 7y	Myopia at 15y
Birth		0.500	0.610
Birth	males	0.500	0.500
Birth	females	0.500	0.500
7		0.638	0.500
7	males	0.739	0.500
7	females	0.733	0.500
15–17		0.500	0.556
15–17	males	0.500	0.500
15–17	females	0.681	0.553

Capacity is measured as area under the receiver operating characteristic curve (AUC).

## Discussion

The design of the Avon Longitudinal Study of Parents and Children (ALSPAC) has allowed us to study intergenerational environmental influences on developmental variation in a general population context. Our recent approach to investigate the effects of early life experiences on subsequent generations has focused on grandmaternal smoking in pregnancy as the specific exposure of interest, testing possible associations with a range of developmental traits measured over time in the study children. Our findings so far^22,24–27,29^ have demonstrated some common features of what are potentially ‘intergenerational responses’; and these features have informed our prior hypotheses in the present study of myopia. We selected myopia as a phenotypic outcome of interest due to its rising prevalence and high heritability, little of which is currently explained by genetic studies. We have found a strong negative association between the paternal grandmother smoking in pregnancy and her grandson’s chance of developing myopia by the age of 7 years. There was a less strong, but again a negative, association with the maternal grandmother smoking in pregnancy and myopia in her grandchildren. And in line with our prior hypothesis, these associations are more evident when the mother herself did not smoke in her pregnancy with the relevant study child. Importantly however, no associations were observed with later–onset myopia arising between 7 and 15, which suggests that different mechanisms may influence earlier-onset vs later-onset myopia. Consistent with this, we observed distinct associations with DNA methylation throughout childhood. In general, methylation associations with myopia at 7 were much stronger (Table 7) and more numerous (Table 5) than were associations with myopia at 15 in spite of the much smaller numbers of individuals with myopia at 7. Little can be said about direction of effect, i.e. whether DNA methylation differences played a role in myopia or myopia a role in DNA methylation differences. However, the existence of associations observed in cord blood suggest that at least some methylation differences were not environmentally induced in childhood.

One association between myopia at 7 with DNA methylation was near the *PAX6* gene (Table 5; Fig. 1). Genetic variants in the *PAX6* gene have been linked to extreme myopia^30^ and mutations of the gene have been linked to ocular and neurodevelopmental disorders^31–35^.

To our knowledge there are no other studies that have looked at the relationship between grandparental smoking in pregnancy and myopia risk. There have, however, been investigations that look at parental smoking in general and its association with refractive error in their young child. Two papers have reported protective associations^36,37^. However a meta-analysis of 12,874 children including these two studies together with the large MPEDS study^38^ found no overall association between maternal smoking and myopia^39^. There is therefore controversy in the existing literature regarding whether or not prenatal smoking affects childhood myopia and our results suggest some of these earlier findings may be confounded by tobacco exposure from the previous generation.

In terms of potential intergenerational mediating mechanisms for our observations, it is appropriate to note the reproductive differences between the maternal and paternal lines. By the third trimester a female fetus (the study mother) will already have ovaries with oocytes proceeding through meiosis; these will contain paired (and recombining) grandparental chromosomes destined for the grandchildren (the study child). Thus, tobacco exposure could directly impact the study child’s chromosomes, e.g. with persisting epigenetic alterations or by causing DNA damage (see below). Furthermore, there are several other potential intergenerational transmission routes for the exposure effects from the mother, including mitochondrial inheritance, cross-placental transfer of biochemical signals, or via the microbiome and breast milk. On the other hand, paternal transmission of prenatal tobacco exposure effects is less confounded; it is either carried by sperm or seminal fluid. For a male fetus (the study father) the germline does not proceed to meiosis until puberty. Thus transmission of his fetal exposure effects to his own children will be likely to be more indirect, with exposure information held in somatic (germline) cells or as induced ncRNAs that ultimately can be transferred to his mature sperm^21^. With either parent, the traditional challenge to direct transmission of induced epigenetic alterations such as DNA methylation between generations has been the existence of two rounds of genome-wide demethylation. These occur early in embryological development in the primordial germ cells and again after conception in the pre-implantation embryo. As noted above, either grandmother smoking throughout pregnancy will expose their fetus beyond the primordial germ cell stage, but there is still the issue of demethylation in the preimplantation stage. Classically imprinted genes and their control regions are protected against this latter demethylation, and there is now evidence in humans that nearly 2000 gene loci (termed ‘escapees’) avoid full demethylation between generations, over and above numerous DNA repeat sequences that are ‘escapees’^40^.

Of interest in terms of imprinted genes and myopia is RASGRF1. This guanine nucleotide exchange factor is an important regulator of intracellular signaling and neural plasticity in the brain. Clearly showing genomic imprinting in rodents and pigs, with brain expression only from the paternally-derived chromosome, there is uncertainty about the imprinting status of the *RASGRF1* gene in humans^41,42^. A more recent study found that there are more imprinted domains in the human placenta than in somatic tissues, and found *RASGRF1* to be paternally expressed in human placenta and that this is controlled by a maternally methylated DMR^43^. Intriguingly we observed an association of DNA methylation with myopia at 7 at a CpG site within 100 bp of a *RASGRF1* transcription start site. However, we cannot conclude too much from this and await further work to determine the true relevance of this association to myopia.

It is noteworthy that we found a marked protective effect against myopia in the grandchildren when they were aged 7, but no effect at all when looking at myopia that developed between the ages of 7 and 15. This suggests that different mechanisms control earlier-onset myopia and later-onset myopia, the former being susceptible to intergenerational effects of smoking. A recent review has used statistical models to argue that myopia appearing before the age of 6 is rare and represents either a syndromic condition or an impairment in the emmetropization mechanism/s, whilst the majority of cases of myopia that occur after this age have a substantial environmental etiology, albeit with genetically-determined variations in susceptibility^44^. Some genetic variants that cause rare syndromes involving myopia, generally in early life, are also reported in GWAS studies of non-syndromic myopia in general populations^45^. Thus early-onset myopia may be caused by a less pathogenic variant of genes that can rarely cause syndromic myopia or by failures in the emmetropization process.

Why might grandmaternal smoking be associated with a *reduced* prevalence of early myopia? The first point to make is that our analyses do not support an explanation in terms of socio-economic position (SEP). If lower SEP grandmothers were more likely to smoke during pregnancy and lower SEP was protective against myopia (since higher education is linked to myopia), then it would be difficult to say whether the cause of myopia was education or grandparental smoking. However, we show that grandparental education and social group are not associated with myopia and that adjustment for these variables does not affect the associations of smoking with myopia. Smoking is an established cause of DNA damage of various kinds including the effects on sperm^46^. The DNA damage response system results in the DNA in the nucleus being less tethered/restrained, with this increased mobility facilitating access to DNA repair complexes^47^. This ‘mobility’ in turn may compromise the control of DNA repeat sequences and transposable elements by DNA methylation and repressive chromatin states in the germline and in turn in the emerging early embryo of the next generation. It is perhaps this altered genomic state, rather than tobacco exposure itself, that contributes to the lessening of the developmental responses that are currently leading to early-onset myopia.

### Strengths and Limitations

There are a number of limitations to the study. (i) As we showed in Table 1, the children whose parents or grandparents had a history of smoking were significantly less likely to attend for examination of vision at age 7. Not only were the children of smokers less likely to attend, but this was particularly true of heavy smokers. This bias precludes the use of strategies to impute missing data, and it may also bias the interpretation of the results. However, it is difficult to hypothesize how that might nullify our results unless the smoking parents whose children did not attend were almost all myopic at age 7. (ii) The categorization of myopia at age 7 is limited by inaccuracies in the method and some children may have been misclassified. (iii) Although the DNA methylation analysis suggests that DNA methylation is better for predicting myopia at 7 than at 15, we should point out that the number of individuals with myopia at 7 is quite small (Supplementary Table 4). Consequently, there is some risk that statistics may be inflated, so this result should be interpreted with caution until it can be replicated in other data. The main result of the DNA methylation analysis, however, is clear – there is little overlap between CpG sites associated with myopia at 7 and those associated with myopia at 15.

The strengths of the study lie in: (i) The fact that the smoking histories of the parents and grandparents were all collected prior to the child being born, and thus prior to any diagnosis of myopia. This precludes any bias resulting from knowledge of the outcome of the child. (ii) Similarly free of bias, the examination of the visual abilities of the children was carried out by staff blind to the parental and grandparental smoking histories, and the selection of DNA for methylation analysis did not depend on either the family history of smoking or the presence of myopia in the child.

## Conclusions

We conclude that: (i) Intergenerational associations observed were confined to early onset myopia at age 7. No associations were observed with myopia developing between 7 and 15 years, thus supporting the view that these two groups have different etiologies. Genome-wide DNA methylation analysis was consistent with this view. (ii) Paternal grandmother smoking in pregnancy was associated with a marked reduction in the prevalence of myopia in her grandsons – and to a lesser extent in her granddaughters. This was most evident when the mother herself did not smoke in pregnancy. (iii) There was a weaker, non-sex-specific, reduction in the prevalence of myopia at 7 if the maternal grandmother had smoked in pregnancy.

These results add further support for the notion that such intergenerational effects are part of human developmental variation and provide a new dimension for research to find potentially modifiable causes of early-onset myopia.

## Methods

The data used in these analyses were collected as part of the Avon Longitudinal Study of Parents and Children (ALSPAC), which was designed to assess the ways in which the environment interacts with the genotype to influence health and development^46–48^. Pregnant women resident in the study area in south-west England with an expected date of delivery between 1^st^ April 1991 and 31^st^ December 1992, were invited to take part. About 80% of the eligible population did so. The initial ALSPAC sample consisted of 14,541 pregnancies; of these initial pregnancies, there was a total of 14,676 fetuses, resulting in 14,062 live births and 13,988 children who were alive at 1 year of age. Information on the cohort parents and their offspring was collected using a variety of methodologies including self-completion questionnaires sent to study mothers, fathers, teachers and the study child, direct examination under standardized conditions, and linkage to educational data from the school system. Please note that the study website contains details of all the data that is available through a fully searchable data dictionary and variable search tool (http://www.bristol.ac.uk/alspac/researchers/our-data/).

Ethical approval for the study was obtained from the ALSPAC Ethics and Law Committee (ALEC; IRB00003312) and the Local Research Ethics Committees^49^. Detailed information on the ways in which confidentiality of the cohort is maintained may be found on the study website: http://www.bristol.ac.uk/alspac/researchers/research-ethics/.

ALEC agreed that consent was implied if questionnaires were returned. Informed written consent was obtained for all biological samples prior to analysis, and for certain invasive procedures during the hands-on assessments (which were optional to attend) from the participant and/or legal guardian^49^. All study methods were performed in accordance with relevant guidelines and regulations. Together with the local Health Services ethics committees ALEC has approved the linkage of the DNA and methylation data to the detailed assessments and other information on the parents and children. Analyses of biological samples including genetic and DNA methylation are only carried out for individuals for whom informed generic consent has been received.

### Assessment of refractive error and classification of myopia

A series of vision assessments were carried out at the ALSPAC research clinics held when the participants were aged approximately 7, 10, 11, 12 and 15 years-old. Refractive error was assessed at each time point using non-cycloplegic autorefraction (Canon R50 instrument, Canon USA Inc., Lake Success, NY). Mean spherical equivalent (MSE) refractive error was calculated using the equation, MSE = sphere power + (0.5 × cylinder power). The refractive error of the participant was taken as the average MSE in the two eyes. At each age (7–15 years), participants were classified as ‘likely myopic’ vs. ‘likely non-myopic’ if their average MSE ≤ −1.00 D. We used as our outcomes (i) myopia at age 7 as this was the largest sample and (ii) myopia at 15 (minus those detected at 7) as the most up-to-date follow up on the participants.

### The exposures

The pregnant study mothers and their partners (F1) were sent a number of questionnaires during pregnancy. These elicited details of their smoking histories and those of their parents, i.e. the study grandparents (F0).

### Possible confounders

Other information collected on the four study grandparents included the years in which they had been born, their ages when the study parent was born, their social group (based on their occupations), their educational qualifications (grouped into the equivalent of O-level (exams taken at about age 16) or higher versus lower levels of qualifications (or none)), ethnic group (grouped as white or other), and (for grandmothers only) parity – i.e. whether the study parent was the first or later birth to that grandmother. Finally, we included social group obtained for each parent based on their occupation. Each of these variables was considered as a potential confounder.

### Self-reported parental vision

Each of the study parents answered questionnaires that enquired for each eye, ‘*How would you rate your sight without glasses?’* The answers they could choose were: *‘always very good; I can't see clearly at a distance; I can't see clearly close up; I can't see much at all’*. From these responses, we derived a binary variable indicating whether or not each parent had a self-reported visual defect for use in the analyses.

### Statistical analyses

For our primary outcome myopia at 7 (early onset) we performed an initial set of logistic regression analyses – first looking at the unadjusted odds ratios (ORs) and 95% confidence intervals (CIs) for each of the features of the grandparents outlined in the possible confounders section above. We selected all variables that were statistically significant at P < 0.10 to include in backward stepwise logistic regression analyses. These analyses were termed adjustment (A). Further adjustment (B) included whether or not the relevant parent had reported reduced visual acuity at near, far or any distance (Supplementary Table 2). The results were then investigated to determine whether there were differences in the results if the mother herself smoked prenatally, and whether there were differences between the results for boys and girls by assessing whether there was overlap between the CIs.

A similar process was undertaken for the children who were myopic at 15 but not at 7. This was not pursued as there were minimal unadjusted associations for this group (Supplementary Table 2).

### DNA methylation

DNA methylation profiles for ALSPAC children were generated at birth from cord blood and in childhood from peripheral blood at ages 7 and 15–17 using the Illumina Infinium HumanMethylation450 BeadChip as part of the Accessible Resource for Integrated Epigenomic Studies (ARIES)^50^. Quality control, preprocessing and normalization of the profiles was performed using the *meffil* R package as previously described^51^. Associations with probable myopia at 7 and myopia at 15 were tested using linear models with DNA methylation as the dependent variable and myopia as an independent variable along with 20 covariates generated using surrogate variable analysis^52^ to adjust for potential known (sex, cell count variation, batch) as well as unknown confounders^53^. P-values were adjusted for multiple tests (~450 K) using Bonferroni correction. DNA methylation outliers were handled by 2.5%-winsorization. Associations were tested using modified *t*-statistics implemented in the *limma* R package^54^. Only associations with autosomal DNA methylation were tested.

The capacity of DNA methylation to predict myopia was assessed by generating DNA methylation predictors of myopia and measuring their performance under 10-fold cross validation. Predictors were generated by fitting elastic net models implemented in the *glmnet* R package^55^ within the training partition of the dataset restricted to the 1000 CpG sites most strongly associated with myopia in the training partition. Models were then applied in the testing partition to produce a DNA methylation ‘score’. The association of the score with myopia was measured as the area under the receiver operating characteristic (AUC) curve. The AUC for the testing partition of each of the 10 folds were combined by taking the mean.

## Supplementary information

Supplementary Information

## Data Availability

In order to preserve confidentiality of the participants, it is important that the ALSPAC access rules are taken into account. The ALSPAC study website contains details of all the data that are available through a fully searchable data dictionary: http://www.bristol.ac.uk/alspac/researchers/our-data/. Data can be obtained by bona fide researchers after application to the ALSPAC Executive Committee (http://www.bristol.ac.uk/alspac/researchers/access/).
