# Author Correction: Grandmothers’ smoking in pregnancy is associated with a reduced prevalence of early-onset myopia

**DOI:** 10.1038/s41598-019-56284-3

**Published:** 2019-12-16

**Authors:** Cathy Williams, Matthew Suderman, Jeremy A. Guggenheim, Genette Ellis, Steve Gregory, Yasmin Iles-Caven, Kate Northstone, Jean Golding, Marcus Pembrey

**Affiliations:** 1 Centre for Academic Child Health, Population Health Sciences, Bristol Medical School, Oakfield House, Oakfield Grove, University of Bristol, Bristol, BS8 2BN UK; 2 MRC Integrative Epidemiology Unit, Bristol Medical School, Oakfield House, Oakfield Grove, University of Bristol, Bristol, BS8 2BN UK; 3 School of Optometry & Vision Sciences, Cardiff University, Maindy Road, Cardiff, CF24 4HQ UK; 4 ALSPAC, Oakfield House, Oakfield Grove, University of Bristol, Bristol, BS8 2BN UK

Correction to: *Scientific Report* 10.1038/s41598-019-51678-9, published online 28 October 2019

This article contained errors.

As a result of an error during the preparation of the manuscript, the original version of this article contained the same figure included twice as Figure 1b and Figure 2. This has now been corrected and the redundant Figure 2 has been removed from both the HTML and PDF versions.

