# Supplementary material for: Grandmothers’ smoking in pregnancy is associated with a reduced prevalence of early-onset myopia
Source: Sci Rep. 2019 Oct 28;9:15413. doi: 10.1038/s41598-019-51678-9 (PMC6817861; doi:10.1038/s41598-019-51678-9)
Supplement: Supplementary file 1 — Supplementary Information [file 41598_2019_51678_MOESM1_ESM.pdf]

Grandmothers' smoking in pregnancy is associated with a reduced prevalence of early-onset myopia

Cathy Williams, Matthew Suderman, Jeremy A. Guggenheim, Genette Ellis, Steve Gregory, Yasmin Iles-Caven, Kate Northstone, Jean Golding, Marcus Pembrey

Supplementary material

Assessment of refractive error and classification of myopia

In a subset of 344 participants whose subjective refractive error at age 15 years (on a date within ±6 months of the research clinic visit) was obtained from their optometrist, this definition based on their age-15 non-cycloplegic estimate had 90% sensitivity and 94% specificity in correctly identifying myopia of at least -0.75 D.[1] However, at the age of 7, the same technique had been predictably less accurate and was only 67% sensitive and 95% specific for myopia of at least -0.75D.[2] At this younger age of 7, we therefore omitted those children who were not found also to be myopic at 10; we assume that the remaining children were 'likely myopic', based on their autorefractor readings. We acknowledge that autorefraction without cycloplegia will have resulted in some misclassification of truly myopic and truly non-myopic individuals, however we refer to children with 'likely myopia' and 'likely non-myopia' as myopic and non-myopic for ease of understanding.

The exposures

If F1 mothers reported that their own mothers (F0) had smoked, they were each asked whether their mothers had smoked when expecting them – they were given the responses yes/no/don't know from which to select. Thus, the parents who replied 'don't know', had a mother who smoked but the parent was unsure whether she had smoked during her pregnancy. We have analyzed these data assuming that these women did smoke during pregnancy. This assumption has been validated by demonstrating that the mean birthweights of this group of study mothers were reduced when compared with those who reported that their mother had definitely not smoked in pregnancy.[3]

1. Guggenheim, J.A., et al. Time outdoors and physical activity as predictors of incident myopia in childhood: A prospective cohort study. *Invest. Ophthalmol. Vis. Sci.* **53**, 2856-2865 (2012).
2. Williams, C., Miller, L., Northstone, K. & Sparrow, J.M. The use of non-cycloplegic autorefraction data in general studies of children's development. *Br. J. Ophthalmol.* **92**(5),723-724 (2008).
3. Miller, L.L., Pembrey, M., Davey Smith, G., Northstone, K. & Golding, J. Is the growth of the fetus of a non-smoking mother influenced by the smoking of either grandmother while pregnant? *PLoS ONE.* **9**(2), e86781 (2014).

Supplementary Table 1. Proportion (n) of children with confirmed myopia at 15 (not at 7) according to features of their grandparents. [P values <0.10 are in bold].

**Variable**	**MGM**	**MGF**	**PGM**	**PGF**
Year of birth				
Pre 1925	17.8% (77)	17.9% (135)	18.3% (85)	17.5% (125)
1925-1929	18.3% (108)	16.8% (123)	16.0% (75)	17.1% (88)
1930-1934	16.7% (152)	15.8% (140)	16.6% (99)	16.3% (89)
1935-1939	16.3% (163)	17.6% (145)	16.6% (89)	17.3% (73)
1940-1944	14.7% (111)	14.6% (72)	13.9% (51)	11.9% (27)
1945+	15.3% (75)	15.1% (39)	21.9% (35)	23.6% (17)
P	0.508	0.649	0.304	0.356
N	4177	3951	2593	2497
Ethnic background				
White	16.2% (709)	16.2% (706)	16.4% (589)	16.3% (582)
Non-white	27.8% (20)	23.8% (19)	25.4% (17)	27.1% (23)
P	**0.010**	**0.074**	**0.052**	**0.009**
N	4451	4434	3663	3657
Education level				
Lower	15.7% (216)	16.1% (236)	16.5% (299)	16.1% (269)
Higher	17.2% (361)	16.9% (532)	18.0% (185)	16.6% (195)
P	0.235	0.565	0.287	0.719
N	3481	3273	2839	2842
Ever smoked				
Yes	17.1% (391)	16.9% (532)	17.2% (341)	16.9% (452)
No	15.8% (329)	16.2% (188)	16.1% (269)	16.4% (119)
P	0.267	0.614	0.387	0.749
N	4367	4313	3655	3402
Age at birth of parent				
<25 years	15.5% (220)	16.4% (116)	17.5% (178)	14.3% (75)
25-34	16.7% (380)	16.3% (383)	16.5% (289)	17.7% (312)
35+	17.6% (86)	17.1% (155)	16.1% (71)	16.9% (135)
P	0.233	0.485	0.454	0.356
N	4177	3951	3210	3089
Parity				
0	17.4% (263)	NA	15.7% (87)	NA
1+	16.0% (478)		16.6% (152)	
P	0.229		0.673	
N	4499		1473	
Smoked prenatally				
Yes	17.5% (252)	NA	17.4% (248)	NA
No	16.0% (465)		16.2% (359)	
P	0.229		0.339	

N	4345		3642	
Social group				
P	0.833	0.364	**0.043**	**0.094**
N	2607	3841	1958	3416

MGM = maternal grandmother; MGF = maternal grandfather; PGM = paternal grandmother; PGF = paternal grandfather

Supplementary Table 2. The associations between parental self-reported vision at the time of the pregnancy and myopia in childhood and adolescence

PARENTAL VISION	MYOPIA AT 7		MYOPIA AT 15, NOT 7	
	%(n)	OR [95% CI]	% (n)	OR [95% CI]
Mother L eye				
Always good	1.2%(49)	1.00 Ref	14.2%(354)	1.00 Ref
Can’t see distance	2.6%(60)	2.21 [1.51, 3.23]	20.2%(285)	1.53 [1.28, 1.81]
Can’t see near	2.1%(6)	1.73 [0.73, 4.07]	13.1%(26)	0.91 [0.60, 1.40]
Can’t see much	4.6%(22)	3.97 [2.38, 6.62]	21.5%(67)	1.65 [1.23, 2.21]
P		< 0.0001		< 0.0001
Mother R eye				
Always good	1.1%(45)	1.00 Ref	14.2%(354)	1.00 Ref
Can’t see distance	2.8%(64)	2.61 [1.78, 3.83]	20.0%(284)	1.51 [1.28, 1.80]
Can’t see near	2.5%(7)	2.31 [1.03, 5.18]	14.2%(26)	1.00 [0.65, 1.54]
Can’t see much	4.5%(21)	4.19 [2.47, 7.09]	22.0%(69)	1.71 [1.28, 2.29]
P		< 0.0001		< 0.0001
Father L eye				
Always good	1.2%(39)	1.00 Ref	13.0%(267)	1.00 Ref
Can’t see distance	2.9%(41)	2.45 [1.57, 3.81]	20.8%(196)	1.76 [1.44, 2.16]
Can’t see near	3.3%(8)	2.83 [1.31, 6.13]	19.2%(30)	1.59 [1.05, 2.42]
Can’t see much	5.1%(15)	4.47 [2.43, 8.20]	28.8%(59)	2.71 [1.95, 3.76]
P		< 0.0001		< 0.0001
Father R eye				
Always good	1.2%(40)	1.00 Ref	12.7%(263)	1.00 Ref
Can’t see distance	2.8%(40)	2.37 [1.53, 3.70]	21.0%(196)	1.83 [1.49, 2.24]
Can’t see near	3.0%(7)	2.48 [1.10, 5.60]	19.2%(28)	1.64 [1.06, 2.52]
Can’t see much	5.6%(15)	4.85 [2.64, 8.90]	31.3%(61)	3.14 [2.26, 4.37]
P		< 0.0001		< 0.0001

Supplementary Table 3. The associations between parental smoking and myopia in childhood and adolescence

PARENTAL SMOKING	MYOPIA AT 7		MYOPIA AT 15, NOT 7	
	%(n)	OR [95% CI]	% (n)	OR [95% CI]
Mother				
Ever smoked				
Yes	1.6%(53)	0.71 [0.54, 1.01]	15.8%(298)	0.91 [0.78, 1.07]
No	2.2%(89)	1.00 Ref	17.1%(452)	1.00 Ref
P		**0.054**		0.267
Smoked pre-pregnancy				
Yes	1.5%(29)	0.70 [0.46,1.05]	14.6%(157)	0.84 [0.69, 1.01]
No	2.1%(114)	1.00 Ref	17.0%(593)	1.00 Ref
P		**0.086**		**0.064**
Smoked 1st trimester				
Yes	1.6%(23)	0.81 [0.52, 1.27]	13.8%(100)	0.78 [0.62, 0.98]
No	2.0%(120)	1.00 Ref	17.0%(650)	1.00 Ref
P		0.366		**0.032**
Smoked mid-pregnancy				
Yes	1.7%(18)	0.83 [0.50, 1.36]	13.7%(76)	0.78 [0.60, 1.01]
No	2.0%(125)	1.00 Ref	16.9%(674)	1.00 Ref
P		0.455		**0.057**
Smoked 3rd trimester				
Yes	1.6%(17)	0.80 [0.48, 1.34]	13.7%(71)	0.78 [0.60, 1.02]
No	2.0%(122)	1.00 Ref	16.9%(650)	1.00 Ref
P		0.404		**0.069**
Passive smoke exposure[a]				
None	2.1%(57)	1.00 Ref	16.9%(289)	1.00 Ref
< 1hr/day	1.3%(7)	0.58 [0.26, 1.27]	15.1%(54)	0.87 [0.64, 1.20]
1+hr/day	2.0%(56)	0.92 [0.64, 1.34]	15.4%(257)	0.90 [0.75, 1.08]
P		0.677		0.240
Partner				
Smoked mid-pregnancy				
Yes	1.7%(39)	0.84 [0.58, 1.22]	15.1%(199)	0.87 [0.73, 1.04]
No	2.0%(98)	1.00 Ref	17.0%(525)	1.00 Ref
P		0.351		0.134

[a]In pregnancy

P values <0.10 are in bold

Supplementary Table 4. Numbers of study children with DNA methylation profiles.

	Age 7		Age 15	
**Sample**	**none**	**myopia**	**none**	**myopia**
cord blood	840	22	636	121
males	409	12	312	52
females	431	10	324	69
age 7 blood	897	23	680	137
males	442	13	343	58
females	455	10	337	79
age 15-17 blood	898	23	685	135
males	432	13	335	56
females	466	10	350	79

Supplementary Table 5. Overlap of DNA methylation profiles between the same myopic individuals.

		Myopia at 7			Myopia at 15		
		birth	age 7	age 15-17	birth	age 7	age 15-17
**Myopia at 7**	birth	22	22	22	0	0	0
	age 7	22	23	23	0	0	0
	age 15-17	22	23	23	0	0	0
**Myopia at 15**	birth	0	0	0	121	119	117
	age 7	0	0	0	119	137	133
	age 15-17	0	0	0	117	133	135

Supplementary Table 6. Myopia gene candidates.

Gene	Rationale	Number of CpG sites
APLP2	Rare variant of large effect. Promoter variant shows GxE effect for time spent reading	44
RASGRF1	Possibly imprinted locus	55
GJD2	Most strongly associated variant in GWAS studies	14
LAMA2	Largest effect size in GWAS studies	43
ZMAT4	Variant shows replicated GxE interaction for time spent reading/education	29
RBFOX1	Variant shows GxE interaction for time spent reading	109
TSPAN10	Implicated in a range of ocular disorders in addition to myopia	13
DRD1	Top GWAS candidate related to dopamine signalling	12
CASC15	Long non-coding RNA (LINC00340) implicated in myopia and astigmatism	73
	**Total CpG sites**	392

Supplementary Figure 1

No myopia at 7y

Myopia at 7y

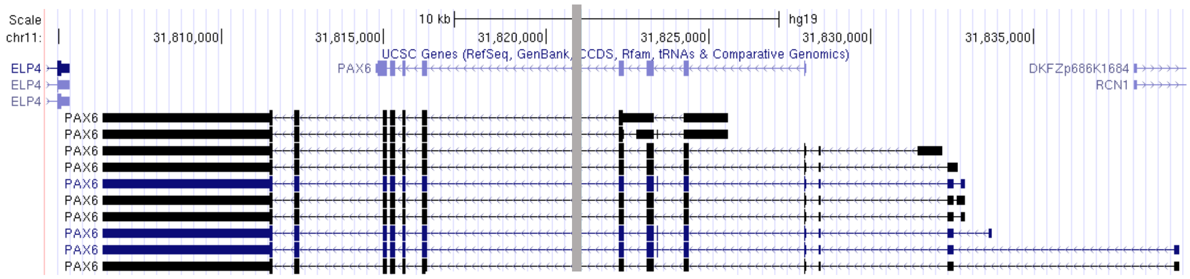

CpG site cg03905867
